# Linking unfolded protein response to inflammation and depression: potential pathologic and therapeutic implications

**DOI:** 10.1038/s41380-018-0241-z

**Published:** 2018-09-13

**Authors:** Matthew II Timberlake, Yogesh Dwivedi

**Affiliations:** Department of Psychiatry and Behavioral Neurobiology, University of Alabama at Birmingham, Birmingham, AL USA

**Keywords:** Depression, Neuroscience

## Abstract

Depression is a devastating mental disorder that affects millions of people worldwide. Inflammation has been shown to be a key factor involved in the underlying pathophysiology of depression and has been shown in a substantial proportion of cases of depression. Changes attributed with morphological deformities and immunomodulation in susceptible regions of the depressed brain raised the possibility of altered cellular homeostasis transduced by the intracellular stress response. How emotional stressors can lead to an inflamed brain that directly affects physiology and activity is yet to be fully understood. The unfolded protein response (UPR) has been shown to be active in both models of depression as well as in postmortem brain of depressed individuals. The UPR is the cellular response to stress which results in misfolded proteins. Interestingly, UPR activation is directly linked to both inflammatory cytokine production and Toll-like receptor (TLR) expression. The TLRs are part of the innate immune response which typically reacts to “classic invasions” such as bacteria or viruses as well as trauma. TLRs have also been shown to be upregulated in depression, thus solidifying the connection between inflammation and depression. In this review, we aim to tie the UPR–TLR response and depression, and describe the implications of such an association. We also propose future directions for their role in treatment for depression.

## Introduction

Depression is a serious mental illness, that affects 300 million people worldwide – the female to male ratio being 1.7:1. [1, 2]. Considering the overall disability and sufferings caused by this mental disorder, it has been predicted to be the leading cause of global disease burden by 2030 [3]. Further, increased suicidality associated with lifetime depression has been reported to be highly comorbid over other psychiatric conditions [4]. Therefore, a strong need to understand the neurobiology associated with this disorder is long-standing. While much of the underlying physiology of depression is well characterized, a deeper insight related to cellular and molecular abnormalities associated with this depression can help to ameliorate the pathological conditions by optimizing more diverse treatment options. Hence, the focus of this review is centered on the molecular mechanisms that underlie the physiology of stress and inflammatory responses in constituting the behavioral deficits associated with major depression. Moreover, the emphasis has been given to understand the involvement of unfolded protein response (UPR) and Toll-like receptor (TLR) modulation in causing depression-associated inflammatory changes at the central nervous system.

As it stands, there are many neurobiological aspects associated with depression from monoamine dysfunctionality [5–8] to neurotrophic deficiency [9] and to inflammation and its many pathways which impact several cellular components in brain resulting in overall neuromodulation [10]. When considering future treatment for depression, it should imperative to study systems that encompass all of these areas in one way or another. Our lab proposes that the UPR may be an unexplored key in regulating or agitating much of this physiology associated with depression. Our previous reports in a preclinical model of depression have shown noticeable changes in transcription of genes related to UPR in susceptible brain areas such as hippocampus [11, 12]. Zhang et al. [13] have also shown similar UPR-related activity in hippocampus and have suggested that the hippocampus exhibits significant apoptotic activation under restraint stress paradigm. Their findings were further supported by the identification of heightened inflammatory response in those restrained rats where part of the activated inflammatory pathways demonstrated the upregulated expression of key mediators like c-Jun kinase (JNK) and Xbox Binding Protein-1 (XBP-1). Finally, they showed that rats under these paradigms had depressive behaviors [13]. A single-nucleotide polymorphism in the 116C/G gene promotor for XBP-1 has also been identified as a potential risk for bipolar disorder which affected sensitivity to mood stabilizing treatments such as lithium [14]; however, another study found no such link [15]. Increased UPR activity was further shown to be associated with depression in the temporal cortex of subjects who died by suicide. The underlying cause of this induced UPR response represented with increased expression of two endoplasmic reticulum (ER) stress-related proteins, glucose regulated protein 78 kD and 94 kD (GRP78 and GRP94), might have been part of a compensatory mechanism initiated to counter the stress-related neuronal damage [16].

## The unfolded protein response

**Fig. 1:**
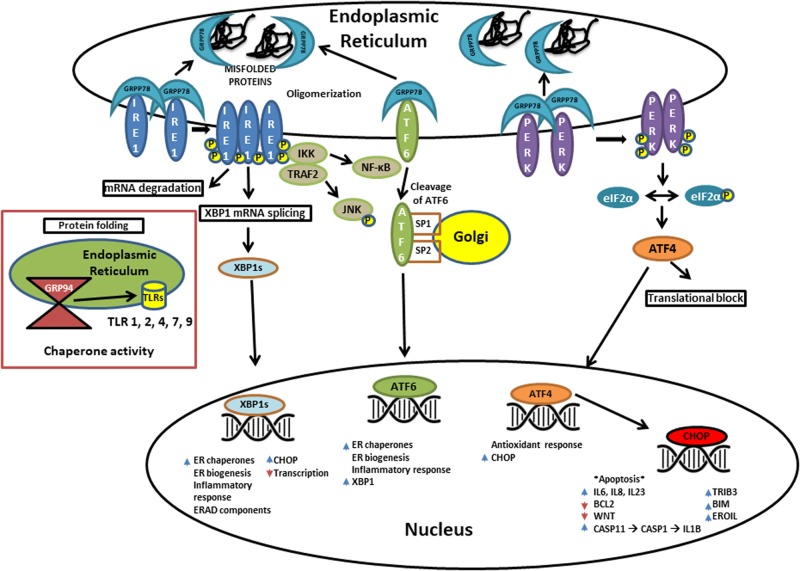
The endoplasmic reticulum is equipped to deal with misfolded proteins with the aims of repairing/stabilizing the proteins, removing proteins that cannot be repaired, or, in the case of extreme overload, to initiate apoptosis. In this way, the UPR is a key regulator of cell fate in the face of stress. As shown above, the three resident sensory proteins are ATF6, IRE1, and PERK. Each of these is inhibited from dissociating (in the case of ATF6) or dimerizing/oligomerizing (IRE1 and PERK); GRP78/BiP is responsible for this inhibition. When a misfolded protein is present in the ER, GRP78 preferentially binds to it as a form of stabilization. At this point, the downstream pathways are initiated which result in increased inflammation, protein translation blocking, and apoptosis. Further, UPR activity is directly related to increased Toll-like receptor folding as GRP94 folds the TLRs. This gives further connection to pro-inflammatory cytokine production and thus inflammation

The UPR is an evolutionarily conserved adaptive mechanism that responds to misfolded proteins which accumulate in the ER under several physiological perturbations. Protein misfolding can occur due to homeostatic disturbance in ER. This intracellular disturbance could be mediated by several intrinsic changes like alteration in cellular metabolites (levels of glucose), cellular environment (such as pH and temperature), and effect mediated by extrinsic factors like administration of pharmacological agents such as tunicamycin (an antibiotic released by *Streptomyces lysosuperificus*). A diagram showing UPR and its downstream pathways is depicted in Fig. 1. Three resident stress sensors, protein kinase RNA-like ER kinase (PERK), activating transcription factor 6 (ATF6), and inositol-requiring enzyme-1 (IRE1), in ER primarily govern the UPR activation to restore the ER homeostasis upon sensing cellular signals generated by unfolded or misfolded protein repertoires. The activated UPR involves a series of intracellular signaling pathways to restore the cellular instability elicited by ER stress response. The experimental data have demonstrated the dissociation of ER chaperone GRP78, or binding immunoglobulin protein (BiP), from any of the three sensor proteins in the ER under stress to initiate the downstream signaling cascade [17]. Once detached, the sensors are then free to perform their previously inhibited intracellular activity. Besides fostering the ER homeostasis under abnormal proteomic load, the cellular pathways associated with UPR signaling play a pivotal role in mounting immune response. The UPR-stimulated immune activation acts as cellular salvage against ER stress which may either restore ER homeostasis or activate pro-apoptotic pathways [18]. Activation of the UPR stimulates upregulation of chaperone molecules such as GRP78/BiP and GRP94, which then initiate and maintain inflammation both through upregulation of TLRs and subsequently transcription/upregulation of cytokine genes [19] as well as other inflammatory-centered pathways [20, 21].

Studies have demonstrated that the apoptotic branch of the UPR results from PERK dimerization/oligomerization [22, 23]. When released by BiP, PERK dimerizes and is activated, which, in turn, phosphorylates eukaryotic initiation factor 2 alpha kinase (eIF2α). This ultimately leads to the preferential transcription of activating transcription factor 4 (ATF4) and translation of ATF4 messenger RNA despite reducing overall translation and protein synthesis. Like XBP-1, ATF4 is a master regulator and a primary determinant of cell survival versus pro-apoptotic signaling [22]. When ATF4 is being continuously transcribed and translated, other pro-survival proteins show reduced expression. It has been suggested that ATF4 promotes the activation of apoptotic pathways by stimulating the C/EBP homologous protein (CHOP) expression. The role of CHOP in inducing cellular death under ER stress has been tested in homozygous CHOP (Chop−/−) knockout mice. During prolonged stress CHOP-deficient mice have shown partial resistance to ER-induced stress [24]. CHOP has been shown to induce ERO1α (an oxidase which catalyzes oxidation in the ER lumen) and BIM, while it simultaneously decreases the expression of BCL-2, which is a cellular protective protein [13, 25, 26]. Therefore, activation of CHOP as downstream target of ATF4 and sustained signaling through PERK–ATF4–CHOP axis leads to a pro-apoptotic environment, thus inducing programmable cell death under irreversible cellular damage. A recent study by Yi et al. [27], using rodent model of stress, has shown hypothalamic neuronal injury due to variable amount of stress which was found to be programmed by a series of molecular changes associated with ER stress. The rats were restrained by 8 h of forced ice water swimming which resulted in significantly decreased GRP78 expression reciprocated with a significant increase in ATF4 and CHOP protein expression. The neuronal damages associated with PERK–ATF4–CHOP pathway could have been the result of altered hypothalamus–pituitary–adrenal axis functions in hypothalamic center of those rats impaired by chronic psychological stress [27].

## Role of UPR in inflammation

Besides several other risk factors which can act as pro-depressant, psychosocial stress has been considered undoubtedly as a primal component in precipitating depressive behavior. However, developing inflammatory response was found to be equally contributed by persistent stress exposure and lead us to believe the existence of single causal factor shared by both depression and inflammation. Inflammation impacts many physiological components in the healthy and depressed brain; namely, the metabolic pathways involved in monoamine production including serotonin, noradrenaline, and dopamine, as well as excitatory amino acid glutamate [28]. For example, tetrahydrobiopterin (BH4) has been shown to be inversely related to the levels of interleukin-6 (IL-6) in the cerebrospinal fluid (CSF). Also, BH4 is a key enzyme (co-factor) in the activities of tyrosine hydroxylase and tryptophan hydroxylase [29], the rate-limiting enzymes required for the synthesis of the catecholamine and serotonin neurotransmitters, and has been shown to be correlated with both levels of CSF dopamine and depressive symptoms [28]. Interestingly, cytokines themselves are sufficient to elicit a depressed behavior [30]. Broadly, the cytokines are either pro-inflammatory or anti-inflammatory in nature. IL-1β, IL-6, interferons (IFNs) and tumor necrosis factors (TNFs) are of particular interest in the context of depression as they have impacts on the function of stress physiology, neurotrophic factor production, and downstream impacts on mood and emotional behavior like anxiety, motor activity, motivation, and reward [30–32]. A few postmortem brain studies suggest that expression of innate immune responders (IL-1β, IL-6, TNF-α) and TLRs are altered in postmortem brain of suicide victims [33–35], which includes suicide subjects with major depression. On the other hand, more robust changes in neuroinflammation has been found when glial cell activation were studied in vulnerable brain areas including frontal cortex, anterior cingulate cortex (ACC), and thalamus of subjects affected by major depression [35, 36]. These findings have recently been replicated in a large cohort of patients [37, 38]. The glial cell activation has been supported by neuroimaging studies where changes associated with translocator protein density was measured by distribution volume (TSPO VT) in activated microglia (prefrontal cortex, ACC, and insula) of depressed patients [39]. This TSPO VT serves as a measure for increased neuroinflammation in the brain.

As introduced in previous sections, the UPR has been introduced in reference to its role in regulating and triggering the inflammatory response. For example, it has been shown that CHOP carries the most critical responsibility in inducing caspase-11 and IL-1β under lipopolysaccharide (LPS)-induced inflammation conditions [40]. This study further showed that animals with knockouts of *CHOP* did not induce caspase-11 or IL-1β. CHOP is also important in the production of IL-23 in dendrites [41]. IL-23 plays an important role in the innate immune response, usually in the face of infection. The enhanced activity of CHOP in models of depression provides a stronger connection between the UPR and inflammation [12, 13].

CHOP is not the only part of the UPR system involved in inflammation. IRE1 activation under induced UPR pathway can also lead to a cross-talk in glycogen synthase kinase-3 (GSK3) and XBP-1-regulated cytokine production and thus inflammatory response [42]; GSK3 has been associated with several mood disorders like depression and bipolar disorder [43]. Specifically, it has been shown that IRE1α differently regulates pro-inflammatory cytokines (e.g., IL-1β and TNF-α) through the activities of both GSK3β and XBP-1 [42]. Converging report from other studies have even shown the regulatory effect of activated IRE1 on the autonomous transcription of pro-inflammatory cytokine by means of the IκB kinase (IKK)–nuclear factor kappa-light-chain-enhancer of activated B cells (NF-κB) pathway as well as the JNK–AP1 (activator protein-1) pathway [44]. To further link the UPR to inflammation, GRP94, a chaperone that is upregulated in response to misfolded proteins, has been shown as a master regulator of TLRs [45]. Recently, our lab has shown strong interaction between the UPR and inflammation via activated TLRs. We showed that there is an increased protein–protein interaction between GRP94 and TLRs 2, 4, 7, and 9 [12], which have been previously associated with depression [33]. GRP94, specifically, is important in folding TLRs which are closely involved in mediating the inflammatory response. Our findings not only confirmed the interaction between GRP94 and TLRs, but also demonstrated a significant increase in TLRs 2, 4, 7, and 9 expression at both the transcriptional and translational levels in the hippocampus of restraint rats. In essence, this strengthens the relationship between TLR activity and depressive symptoms while simultaneously linking the unfolded protein response to TLR, and thus inflammatory activity and expression. Several other studies in the past besides our report have implicated the involvement of genes such as calreticulin (CALR), Bax inhibitor 1 (BAX), GSK3β, interferon-gamma (IFNG), and TNF-α in promoting cross-talk with various candidates from UPR pathways [43, 46–50] under affective abnormalities such as stress paradigms in vivo or application of UPR-inducing agents (like tunicamycin) in cell lines. The immunomodulatory effect of stress in activating ER stress response and UPR gene transcription was also tested in depressed patients where a significant change in expression was noticed for BiP, CHOP, ER degradation enhancing mannosidase-like protein, and the splice variant of XBP genes in leukocyte-derived RNA samples from 86 participants [51]. This confirms the findings in preclinical animal models that the UPR activity is actively involved in the patient population. The pitfall in this experiment is that these PCR data were taken from leukocyte-derived RNA. This does not allow for the study of individual brain regions, but it does, as a proof of concept, prove that there is a global increase in ER stress-related gene transcription.

## TLRs and UPR

**Fig. 2:**
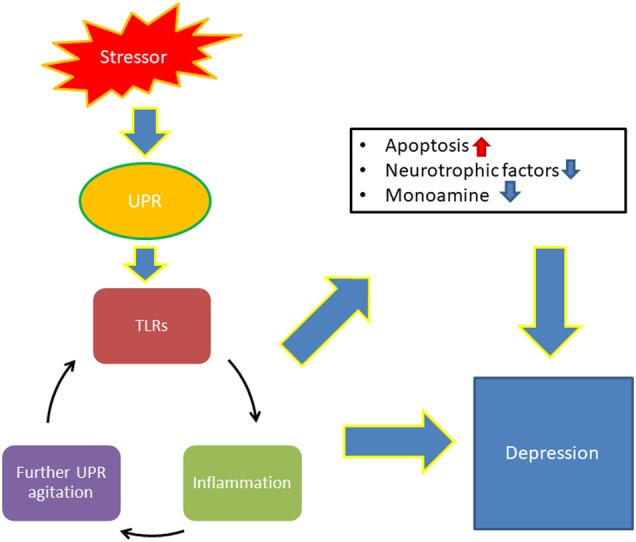
While cellular stressors are typically physical in nature, recent data suggest that psychological stress is sufficient to activate the UPR. Here, we propose that the UPR is vitally important in regulating the underlying pathophysiology associated with major depression

TLRs are an important component of the innate immune response. They are receptors that recognize a host of infectious agents by detecting bacterial walls, flagella, LPS, and RNA and DNA associated with viruses. Once a TLR is activated, they bind with an adaptor protein, myeloid differentiation primary response 88 (MyD88), which then initiates downstream cascades that act on pro-inflammatory cytokines such as ILs, TNFs, and IFNs [42]. Interestingly, despite being a non-pathological disorder, TLRs have been shown to be upregulated in the brain of depressed individuals [33, 52], namely TLRs 2–5, 7, and 9. Further, these TLRs show a diminished expression when treated with antidepressants [53]. To tie this to the UPR, GRP94 is actually responsible for folding and chaperoning all of the above TLRs except TLR3 and 5 [45]. We suggest that when an emotionally salient region of the brain, such as hippocampus, is under psychological stress, it eventually initiates the unfolded protein response. Coinciding with this, TLRs are upregulated as if the cells are under a stressed, physical, or infectious assault. TLRs not only react and message their downstream, pro-inflammatory cascades, but their enhanced expression, as well as the increased presence of heat shock proteins like GRP78 and GRP94, also agitates TLR responses for activation [20, 54]. This generates a continuous loop where the UPR leads to more chaperones, the chaperones activate the TLRs, and the TLRs initiate their pro-inflammatory environment, which further agitates the UPR (Fig. 2). We hypothesize that this is a central part of the pro-inflammatory aspect of depression that couples with the UPR and its damaging effects on neural circuitry. Further, a perpetually activated UPR can result in apoptotic signaling. This may contribute to neuronal atrophy and amplify the effects of decreased brain-derived neurotrophic factor as has previously been reported [55].

## Present and potential treatments for depression

Current common practice is to treat depression with the frontline medications such as select serotonin reuptake inhibitors, serotonin–norepinephrine reuptake inhibitors, and tricyclics. Doses and type of antidepressants vary depending on the side-effect profiles. However, 10–30% of patients do not respond to these treatments and a further third of those have a relapse in depressive symptomology [56]. Interestingly, there is evidence that TLRs respond to antidepressants; however, there is no study to date that has looked at the effects of antidepressant treatment on the UPR. With the present knowledge of the UPR presence and contribution to depression as well as the resulting inflammatory, and potentially apoptotic (i.e. neurodegenerative), contribution by the UPR and its downstream activity, we suggest that future research should more specifically target the UPR as well as TLR-mediated inflammation.

## Drugs targeting the UPR

The UPR holds a host of potential targets for pharmaceutical intervention, from the chaperones to the inflammatory and degenerative arms of UPR activity. The compound azoramide, a drug which has been proposed for diabetic treatment, along with the GRP78 inducer X (drug designed to enhance the expression of GRP78) have been shown to reduce ER stress by enhancing folding capacity [57]. Another target might be XBP-1 and its downstream activity. β-Asarone has been shown to inhibit this pathway and improve symptoms in Parkinson’s disease rat model [58]. Other methods that target XBP-1, at present, are far more theoretical in terms of practical application. Specifically, research has focused on gene editing by either enhancing or diminishing the expression of XBP-1 by the use of viral vectors. While this is an excellent tool for studying specific properties of a disease state, at present, it does not translate to medical intervention.

Much of the research into targeting the UPR has been aimed at the PERK–EIF2–ATF4–CHOP pathway and largely in the context of neurodegenerative disorders like Parkinson’s disease (PD) and Alzheimer’s disease (AD). Some of this work remains theoretical with the aforementioned genetic manipulation, while some of it is more applicable. For example, the compound GSK2606414 is a PERK-specific inhibitor which counteracts the CHOP-related apoptosis; this has largely been studied in PD models with the intent of halting neurodegeneration [59]. Another compound, integrated stress response inhibitor (ISRIB), prevents PERK activation and inhibits eIF2α phosphorylation. Further, this compound was reported to enhance cognitive functions in rodents by diminishing ATF4 as well as *CHOP* expression [60]. It also readily crosses the blood–brain barrier. Compounds like these may actually help some of the cognitive and emotional impairment associated with depression as well as blunt the physiological responses that are the result of UPR activation.

One of the most promising compounds is trazodone hydrochloride, which falls under the classification of a serotonin antagonist and reuptake inhibitor, typically used for their hypnotic properties in conjunction with their reuptake inhibition in depression. Trazodone has been shown to reduce ATF4 levels without affecting eIF2α in cell culture (CHO-KI CHOP:luciferase cells and Chinese hamster ovary (CHO); HEK293 cells, and mouse neuroblastoma N2A cells) that were treated with tunicamycin, suggesting that it works downstream of PERK and eIF2α phosphorylation [61]. This, in effect, halts the global protein inhibition that ATF4 is known to cause. It does not, however, affect other branches of the UPR such as XBP-1 or chaperone expression. This seems like a fine tradeoff if some of the underlying physiology of depression can be attributed to UPR-mediated apoptosis and protein synthesis blocking. Thorough preclinical and clinical studies are needed to fully understand the dosing and duration of treatment given that this compound at current prescribing doses for 6 weeks has not distinguished itself in the clinic. Interestingly, this drug is currently being investigated as a potential therapeutic in neurodegenerative disorders like AD, PD, and prion diseases [61, 62].

TLRs seem to respond well to antidepressants [53]; however, there are a plethora of agents that target specific TLRs and block their downstream pro-inflammatory activities. TLR2 activity can be blocked by the compound “C_29_” [63] or OPN-305 [64]. TLR4 is targeted by the compounds SPA_4_ [65], Eritoran [65], TAK0242 [66], and NI-0101 [67]; the latter three have been abandoned at stage 3 clinical trials as they did not improve symptoms from sepsis or mucosal healing in colitis. They may still serve as therapeutic options for depression. A host of compounds which target the TLR signaling pathway in their relation to cystic fibrosis have been reviewed [68]. Since TLRs regulate much of the pro-inflammatory environment of cells, such compounds may have a therapeutic effect in the context of depression and could be used along with traditional treatment in the cases of severe depression.

## Challenges for therapeutic development

One major drawback to drug usage and development is specificity and accessibility of the blood–brain barrier. Future research may make use of therapeutic agents that are applied directly to a UPR-affected region of the brain (e.g., the hippocampus, amygdala, or prefrontal cortex); however, the researcher should keep in mind the translation to clinical testing. That is, at present, most medication is delivered via intravenous injection or by oral means. Also, only after metabolism, a percentage of the drug is transferred throughout the body. Then, if small enough, it crosses the blood–brain barrier. The problem here is the ubiquitous nature of the UPR and its components. Being that it is an ancient, evolutionarily conserved process, every cell in the body has a stress response and reaction system, the UPR. Hence, when proposing pharmacological intervention, this should be kept in mind. A global knockdown of chaperones, GRP78/BiP for example, would result in global activation of the UPR due to its negative-regulatory nature [69]. As stated before, while more theoretical applications certainly seem useful in understanding basic biology, gene editing may not be appropriate for clinical translation as such therapy would only be effective in a local tissue, thus making it inaccessible to oral-intake medications. Further, it is simply not feasible to propose a therapy in a specific region of the brain.

In the same way, targeting the TLR expressions has negative drawbacks as well. Namely, a global knockdown of these important inflammatory regulators could result in a higher susceptibility to infection [70]. For this reason, when selecting therapeutic agents that target TLRs, one should carefully monitor the patient and any signs of external infection. An actual infection may result in a relapse or compounding of depressive behavior as the inflammatory response to such infections elicits the previously described “sick behavior.” Like the UPR, TLRs are universally expressed and thus difficult to directly treat. Inhibiting targets like MyD88 or specific TLRs can be specifically administered to the emotional regions of interest.

## Conclusion

Depression is a multifaceted and complicated disease with many underlying physiological aspects as well as psychological, genetic, and social aspects. While the standard treatment works well for many individuals, many still are not affected. It is our hypothesis that the UPR and TLR inflammatory pathways contribute largely to underlying physiology and cause more severe depression symptoms. It is vital to understand how and to what extent the UPR plays a role in depression. Future studies should be aimed at understanding its role and emphasizing treatment that focuses on key branches of this system. Trazadone and ISRIB seem to hold promise for treating severe depression as they target specific portions of UPR physiology. In the case of trazodone, which has monoamine reuptake inhibition property, it can help ameliorate some of the psychological duress. Future studies should also focus on the inflammatory environment of the brain, specifically in emotionally salient regions. While there are many routes to do so, we provided research directed toward TLR mediation. Some vulnerability may be present when targeting inflammation such as susceptibility to infection. For this reason, anti-inflammatory research should aim to target areas of the brain as specifically as possible.
